# The effect of work-family conflict on staff nurses’ job performance: the mediating role of emotional intelligence

**DOI:** 10.1186/s12912-025-03280-w

**Published:** 2025-05-30

**Authors:** Nouf Afit Aldhafeeri, Ebtsam Aly Abou Hashish, Hanaa M. Abo Shereda

**Affiliations:** 1 College of Nursing, King Saud bin Abdulaziz University for Health Sciences, Riyadh, Saudi Arabia; 2 King Abdullah International Medical Research Center (KAIMRC), Riyadh, Saudi Arabia; 3 College of Nursing, King Saud bin Abdul-Aziz University for Health Sciences, Jeddah, Saudi Arabia; 4 King Abdullah International Medical Research Center, Jeddah, Saudi Arabia; 5 Faculty of Nursing, Alexandria University, Alexandria, Egypt; 6 Psychiatric Mental Health Nursing Department, Faculty of Nursing, Menoufia University, Menoufia, Egypt

**Keywords:** Work-family conflict, Job performance, Emotional intelligence, Nurses, Saudi Arabia, Nursing workforce

## Abstract

**Background:**

Nurses often experience high levels of Work-Family Conflict (WFC) due to the demanding nature of their profession, which can negatively affect their job performance (JP). Emotional Intelligence (EI) has been identified as a factor that helps individuals manage stress and conflicts effectively, potentially mediating the relationship between WFC and JP. However, the interaction between these variables in the context of the nursing profession, especially in Saudi Arabia, has not been fully explored. This study aims to examine the impact of WFC on JP and assess the mediating role of EI in this relationship among staff nurses.

**Methods:**

A descriptive multivariate correlational design was conducted in a Saudi hospital in Riyadh. A convenience sample of 227 nurses was recruited. Three validated instruments: the Work-Family Conflict Scale, the Six-Dimension Scale of Nursing Performance, and the Wong and Law Emotional Intelligence Scale were utilized for data collection. Descriptive and inferential statistics and path analysis were used to analyze the data.

**Results:**

A statistically significant inverse relationship between WFC and JP (β = -0.08, *p* < 0.01) was reported, suggesting that higher levels of WFC are associated with lower JP. However, WFC did not significantly affect EI (β = -0.02, *p* = 0.35). Importantly, EI had a significant positive impact on JP (β = 0.47, *p* < 0.01) and partially mediated the relationship between WFC and JP, explaining 22% of the variance in JP.

**Conclusion:**

WFC negatively affects JP among nurses, but EI acts as a crucial mediator in improving JP despite WFC. Developing EI among nurses could mitigate the adverse effects of WFC and enhance JP. These findings provide significant implications for healthcare administrators and nurse managers aiming to improve nurse performance, retention, well-being, and organizational outcomes.

**Supplementary Information:**

The online version contains supplementary material available at 10.1186/s12912-025-03280-w.

## Introduction

Nursing is a complex profession that involves emotional, physical, and psychological labor. Nurses represent the largest group of frontline professionals in hospitals, playing a critical role in patient care [1, 2]. With females constituting approximately 80% of the global nursing workforce, nursing is predominantly a female-dominated profession [3]. This high representation of women is often accompanied by increased challenges in balancing work and family responsibilities. Female nurses frequently face the dual pressure of managing household duties alongside the requirements of their professional roles, which can result in conflict between work-family (WFC) [3–5].

WFC arises when the pressures of job and familial responsibilities clash, resulting in stress and potential decreases in work performance. In the nursing profession, this issue is extremely significant, where the overlapping demands of work responsibilities and familial demands can lead to burnout, job dissatisfaction, and compromised patient care [5]. Research highlights that WFC negatively affects nurses’ performance by raising stress levels and decreasing job satisfaction [6]. Emotional intelligence refers to being able to recognize and handle individual’s and other emotions, it has been found that the correlation between WFC and JP is mediated by EI, which buffers the stress’s negative effects [7].

Studies revealed that nurses with higher EI are more capable of managing stress, maintaining healthy interpersonal relationships, and coping with the emotional challenges of their roles [8]. For example, research by Karimi et al. [9] showed that nurses who have higher degrees of EI reported higher JP and lower levels of WFC. Additionally, EI has been shown to improve leadership styles and collaboration, making it a key asset in fostering effective teamwork and improving overall workplace outcomes [10, 11].

### Conceptual framework and development of hypotheses

**Fig. 1:**
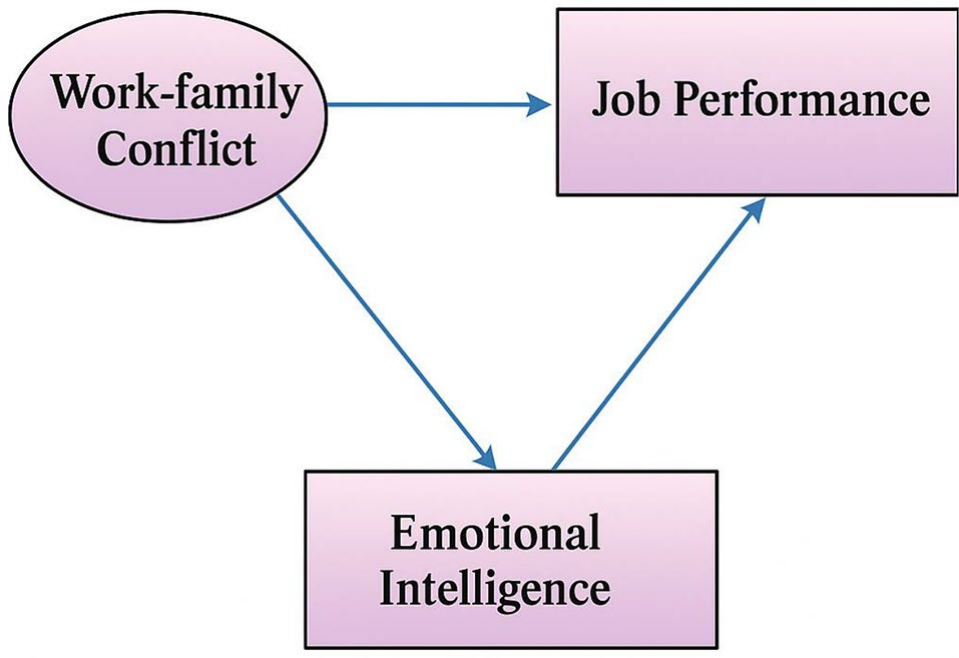
Conceptual Framework

This study conceptualized three key work variables among nurses and the relationships among them: WFC, JP, and EI. See Fig. 1. These factors are integral to understanding how nurses balance the requirements of their work and social lives and how this balance impacts their overall JP.

#### Work-family conflict (WFC)

It is described as a type of inter-role contradiction where the demands and stresses from job and personal domains are not compatible, causing significant strain for the individual [7]. There are two primary dimensions of WFC: work-to-family conflict (WFC) and family-to-work conflict (FWC) [7]. WFC happens in case work requirements interfere with fulfilling familial responsibilities, while FWC happens if family obligations, duties, and stress negatively affect nurses’ focus and efficiency at work, leading to reduced job performance [12, 13]. These two forms of conflict, although distinct, are interconnected. For nurses, these conflicts arise due to a combination of factors such as the heavy responsibilities in both the workplace and the family. Long shifts, emotional labor, and the demanding nature of patient care can lead to WFC, were work pressures spill over into family life, disrupting personal responsibilities. Greenhaus and Beutell [7] emphasized that as the demands in one dimension increase, it gets harder to satisfy the demands of the other, creating a sense of conflict and imbalance. Roy et al. [14] described this as the clash between pressures from different social roles, such as work and family, where expectations in one domain interfere with fulfilling responsibilities in another.

In nursing, where emotional and physical labor is substantial, these conflicts can further exacerbate stress, leading to negative impacts on both nurses’ psychological well-being and JP. Researchers found that nurses who suffer from high levels of WFC or FWC are less able to sustain an adequate balance between work-life, ultimately affecting patients’ care and job satisfaction [12]. Addressing these conflicts is crucial to improving nurses’ psychological health and job performance, specifically in healthcare centers where emotional demands and a lack of staff are common.

#### Work-family conflict (WFC) and job performance (JP)

Job performance (JP) refers to a nurse’s ability to effectively fulfill roles, duties, and responsibilities related to direct patient care in order to meet organizational objectives [15]. In nursing, JP encompasses a broad set of competencies and responsibilities, ranging from leadership to clinical care, and requires a comprehensive approach to performance assessment. This multi-faceted nature of nursing work emphasizes the need for evaluating JP through various dimensions to reflect the complexities of the nursing role [16, 17]. According to Kane [18] and Schwirian [19], JP can be operationally measured across six scopes: critical care activities, leadership, collaboration, evaluation, interpersonal relations, and training and development. These dimensions capture the comprehensive skill set required for effective nursing practice and are crucial for assessing performance in a clinical setting.

Research has demonstrated that there is a substantial relationship linking WFC and nurses’ job performance. Siallagan et al. [20] found that as WFC increases, nurses’ job performance tends to decrease, and vice versa. Similarly, Wang and Tsai [21] confirmed that the two dimensions of (WFC) have a significantly negative impact on JP. In other words, higher levels of either type of conflict result in declining JP among nurses.

The consequences of WFC extend beyond performance. Dilmaghani et al. [5] found that WFC negatively influences nurses’ job satisfaction and quality performance, and may even lead to intentions to leave their jobs. As the requirements in both areas rise, nurses may encounter a decline in their ability to meet the standards of care expected in their roles, which can affect not only individual well-being but also patient outcomes and healthcare quality.

#### Work-family conflict (WFC), job performance (JP), and emotional intelligence (EI)

WFC can significantly affect JP, but certain factors can help mediate or alleviate its impact. EI is essential in managing WFC. Recent findings have demonstrated that greater EI is linked with reduced emotional exhaustion, improved interpersonal relationships, enhanced work satisfaction, and a good correlation with work performance [22–26].

Wong and Law (2002) [27] identified four key components of EI: Self-Emotions Appraisal (SEA), which refers to the capacity to identify and comprehend one’s feelings; The capacity to recognize and comprehend the feelings of others is known as Others-Emotions Appraisal (OEA); Regulation of Emotion (ROE), which is the capacity to control ones’ emotions to improve performance and sustain motivation, Use of Emotion (UOE), which is the capacity to harness feelings for decision-making and problem-solving [28].

In nursing, EI is particularly important due to the emotional demands of the profession. Nurses frequently interact with patients, families, and colleagues in high-stress situations, making emotional management critical. Nurses with greater EI are equipped to handle their feelings and respond effectively to the emotions of others. This ability influences decision-making, patients’ quality of care, and professional relationships [29, 30]. Additionally, EI helps nurses manage work-related stress, address clinical issues, and meet patients’ needs more efficiently.

Research also shows that EI significantly improves leadership styles within healthcare [10, 11]. Leaders with higher EI, especially those with transformational leadership, tend to achieve better organizational outcomes, enhance job performance, and improve workplace dynamics [10, 11]. Conversely, problematic behaviors like inadequate communication and weak collaboration are associated with low EI levels, which can negatively impact team performance and job effectiveness [31, 32]. The role of EI became particularly crucial during the pandemic of COVID-19, when nurses faced unprecedented degrees of stress and burnout [33, 34]. Thus, EI acts as a pivotal function in improving the overall success and job performance of nurses, enhancing both their personal well-being and patient outcomes [29].

According to the conceptualization of the research’s variables, which are WFC, EI, and JP, these hypotheses are postulated:

##### H1

There is a negative relationship between WFC and staff nurses’ JP, as well as their EI.

##### H2

There is a negative relationship between WFC and staff nurses’ JP, as well as their EI.

### Significance of study

Nurses often face significant levels of WFC due to the challenging nature of their work. This conflict arises when the demands of job and family responsibilities clash, leading to increased stress, reduced job satisfaction, and, ultimately, decreased JP. Previous research has shown that WFC is a strong predictor of JP, with increased degrees of conflict leading to decreased degrees of job effectiveness [35]. In healthcare, this issue is particularly prevalent due to increased job demands, staffing shortages, and long working hours, all of which contribute to heightened stress levels for nurses [36, 37]. Furthermore, additional stressors such as a lack of social support, limited opportunities for promotion, and the pressures of caring for critically ill patients exacerbate WFC, leading to negative outcomes for both nurses and their work environments [38].

Despite the clear link between WFC, EI, and JP, there remains a gap in understanding how EI mediates the relationship between WFC and JP in nursing. While earlier research has explored the negative effect of WFC and the benefits of EI, few have examined the specific mechanism by which EI can buffer the adverse effects of WFC on JP among nurses. This research aims to fill that gap by investigating the role of EI as a mediator between WFC and JP among staff nurses.

The importance of this study originates in its capacity to offer valuable, insightful information into how EI can mitigate the harmful effects of WFC on JP in the nursing profession. By focusing on EI as a mediating factor, this study will add a deeper understanding of how emotional competencies can enhance JP, improve nurses’ emotional well-being, and reduce WFC. These findings are particularly important for healthcare administrators and policymakers who aim to create supportive work environments that enhance nurses’ EI, reduce conflict, and promote better JP [39].

Furthermore, this study’s focus on nurses in Saudi Arabia adds an important dimension, as cultural, familial, and professional expectations may influence the relationship between WFC, EI, and JP. By addressing these factors within the unique context of Saudi Arabia, the study provides locally relevant insights, while also contributing to the global literature on nursing JP and EI.

In conclusion, this study adds to existing knowledge by clarifying how EI mediates the relationship between WFC and JP. This knowledge can guide the creation of focused interventions aimed at enhancing nurses’ emotional intelligence, lessening WFC’s detrimental effects, and eventually enhancing healthcare delivery.

### Aim of the study

This study aimed to investigate the impact of work-family conflict (WFC) on staff nurses’ job performance (JP) and to assess the mediating role of emotional intelligence (EI).

## Materials and methods

### Study design, setting, subjects

This study employed a descriptive multivariate correlational design, and it was carried out at a governmental hospital in Riyadh, Saudi Arabia. The hospital provides a full spectrum of healthcare services, from primary to tertiary care. The study population consisted of nurses. Participants were included based on the following inclusion criteria: nurses who have minimum one year of experience and a willingness to take part in the study. Nurses who have less than one year of experience were excluded from the study. The required sample size was calculated using G*Power (3.1) software. A linear multiple regression, fixed model, R² increase analysis was conducted, resulting in a required sample size of 119 nurses. The calculation assumed an effect size of 0.15, a power of 0.95, and an alpha level of 0.05, with 12 predictor variables. To ensure adequate data and account for potential dropouts, the researchers increased the required sample size by 20% to 144 nurses. In this study, the final sample was 227 nurses.

### Data collection procedure and instruments

After receiving ethical approval, the nursing services department was contacted to assist in distributing the online survey to the nursing staff. The ethical approval was attached to the distribution survey along with the consent form. The Microsoft Forms platform was used to disseminate the survey, which consisted of two parts: demographic data and three measurement instruments—the Wong and Law Emotional Intelligence Scale (WLEIS), the Work-Family Conflict Scale, and the Six-Dimension Scale of Nursing Performance.

1. **The demographic section** collected information from participants to describe the sample, including details such as age, marital status with number of children, level of education, and years of experience.
2. **The Work-Family Conflict Scale**, conceptualized to measure the level of conflict between work and family responsibilities [13]. It comprises of 10 items rated on a 5-point Likert scale ranging from strongly disagree to strongly agree. The scale has two subscales: work-to-family conflict and family-to-work conflict. The Cronbach’s alpha for the scale was 0.87 for work-to-family conflict and 0.85 for family-to-work conflict, indicating high reliability [13]. Higher scores represent higher levels of conflict, while lower scores indicate less conflict.
3. **The Wong and Law Emotional Intelligence Scale (WLEIS)** comprises of 16 self-reported items that assess emotional intelligence across four subscales. These include self-emotions appraisal, others-emotions appraisal, use of emotion, and regulation of emotion. The scale uses a 7-point Likert scale (totally disagree to totally agree). The Cronbach’s alpha ranges from 0.80 to 0.89, demonstrating high reliability [27, 40]. Higher scores indicate higher EI, while lower scores indicate lower EI.
4. **The Job Performance Scale**, developed by Schwirian [19], it is a comprehensive tool used to evaluate nurses’ clinical performance, focusing on both the frequency and quality of key job-related behaviors. The scale consists of 52 items divided into six distinct subscales, each addressing critical aspects of nursing performance. These subscales provide a detailed assessment of how nurses fulfill their roles and responsibilities in clinical settings, making it an invaluable tool for understanding nursing competencies. The Leadership subscale, consisting of 5 items, measures a nurse’s ability to lead and manage clinical situations. The Critical Care subscale, comprising 7 items, evaluates nurses’ competence in handling critical care situations. The Teaching/Collaboration subscale, which includes 11 items, assesses nurses’ ability to educate patients, families, and colleagues, as well as their collaboration within healthcare teams. The Planning/Evaluation subscale, consisting of 7 items, focuses on nurses’ ability to plan and evaluate patient care. The Interpersonal Relations/Communications subscale, which has 12 items, examines nurses’ crucial skills for building rapport with patients and families, facilitating teamwork, and ensuring that essential information is shared effectively among healthcare professionals. Finally, the Professional Development subscale, made up of 10 items, measures a nurse’s engagement in activities aimed at enhancing their skills and knowledge [19].

### Validity, reliability, and pilot study

To ensure the validity and reliability of the study instruments, a panel of five academic experts reviewed the content of the scales used in this study. The panel evaluated the relevance, clarity, and cultural appropriateness of the items in the Wong and Law Emotional Intelligence Scale, Work-Family Conflict Scale, and Six-Dimension Scale of Nursing Performance. The internal consistency of these scales was assessed using Cronbach’s alpha, which demonstrated high reliability for each scale: the WLEIS (α = 0.80 to 0.89), the Work-Family Conflict Scale (α = 0.87 for work-to-family conflict and 0.85 for family-to-work conflict), and 0.87 for the Six-Dimension Scale of Nursing Performance. A pilot study was conducted on 10% of the final sample (*n* = 23) to evaluate the clarity, applicability, and completion time of the questionnaire. The pilot data were not included in the final analysis, but the results indicated no need for revisions in the study instruments, confirming their validity and reliability.

### Ethical considerations

Approval for the study was obtained from the KSAU-HS-College of Nursing-Riyadh Research Unit and the King Abdullah International Medical Research Center (KAIMRC) (IRB/2191/22) before the study began. The purpose of the research was briefly explained to all potential participants, emphasizing that there were no risks associated with participation. Participants were informed that they had the freedom to withdraw from the study at any time without facing any penalties. The privacy and confidentiality of participants were strictly maintained. All data collected were anonymized, and no personal information was disclosed. Informed consent was obtained online before participants accessed the questionnaire, ensuring that they were aware of the nature of the study and their rights as participants.

### Statistical analysis

The data collected through Microsoft Forms were exported and imported into SPSS version 26. Descriptive statistics were used to describe the sample. Inferential statistics were applied to answer the research questions. Pearson’s *r* correlation was used to examine the relationships between the study variables. Additionally, multivariate statistics, including path analysis, were employed to predict the mediating effect of emotional intelligence on the relationship between work-family conflict and job performance. Two-tailed p-values of *p* ≤ 0.05 were considered statistically significant.

## Result

### Participants characteristics

The demographic characteristics analysis of the 227 nurses reveals that the majority fall within the 31-40-year age group, representing 53.7% of the sample. The average age of participants is 37.68 years with a standard deviation of 7.89. In terms of experience, nearly half of the participants (51.9%) have more than 10 years of nursing experience, with an average of 10.4 years and a standard deviation of 4.93. The marital status distribution shows that 44.5% of the participants are single, while 44.9% are married with children, reflecting a diverse range of family responsibilities within the workforce.

Educationally, the majority of participants hold a Bachelor of Science in Nursing (BSN) degree (71.4%), while 22.9% have a diploma and 11.5% possess a Master’s degree, indicating a well-educated nursing staff. The majority of participants are staff nurses (86.8%), with 44.9% identified as Staff Nurse I and 41.9% as Staff Nurse II. A smaller proportion of the sample includes unit managers (7.9%), while roles such as nurse specialists and nurse coordinators make up a smaller percentage of the workforce. Table 1.

**Table 1:** Distribution of the study sample by demographic characteristics (N = 227)

Variable	*N*	%		
**Age**				
< 21 years	1	0.4		
21–30 years	39	17.2		
31–40 years	129	53.7		
41–50 years	51	22.5		
> 50 years	20	8.8		
**Marital Status**				
Single	101	44.5		
Married, no kids	23	10.1		
Married with kids	102	44.9		
Widow	5	2.2		
**Educational Level**				
Diploma (less than three years)	52	22.9		
BSN	162	71.4		
Master’s Degree	26	11.5		
**Years of Experience**				
< 1 year	10	4.4		
1–5 years	35	15.4		
6–10 years	77	33.9		
> 10 years	118 51.9			

### Perceived level of nurses’ work-family conflict (WFC), Job performance (JP), emotional intelligence (EI)

**Fig. 2:**
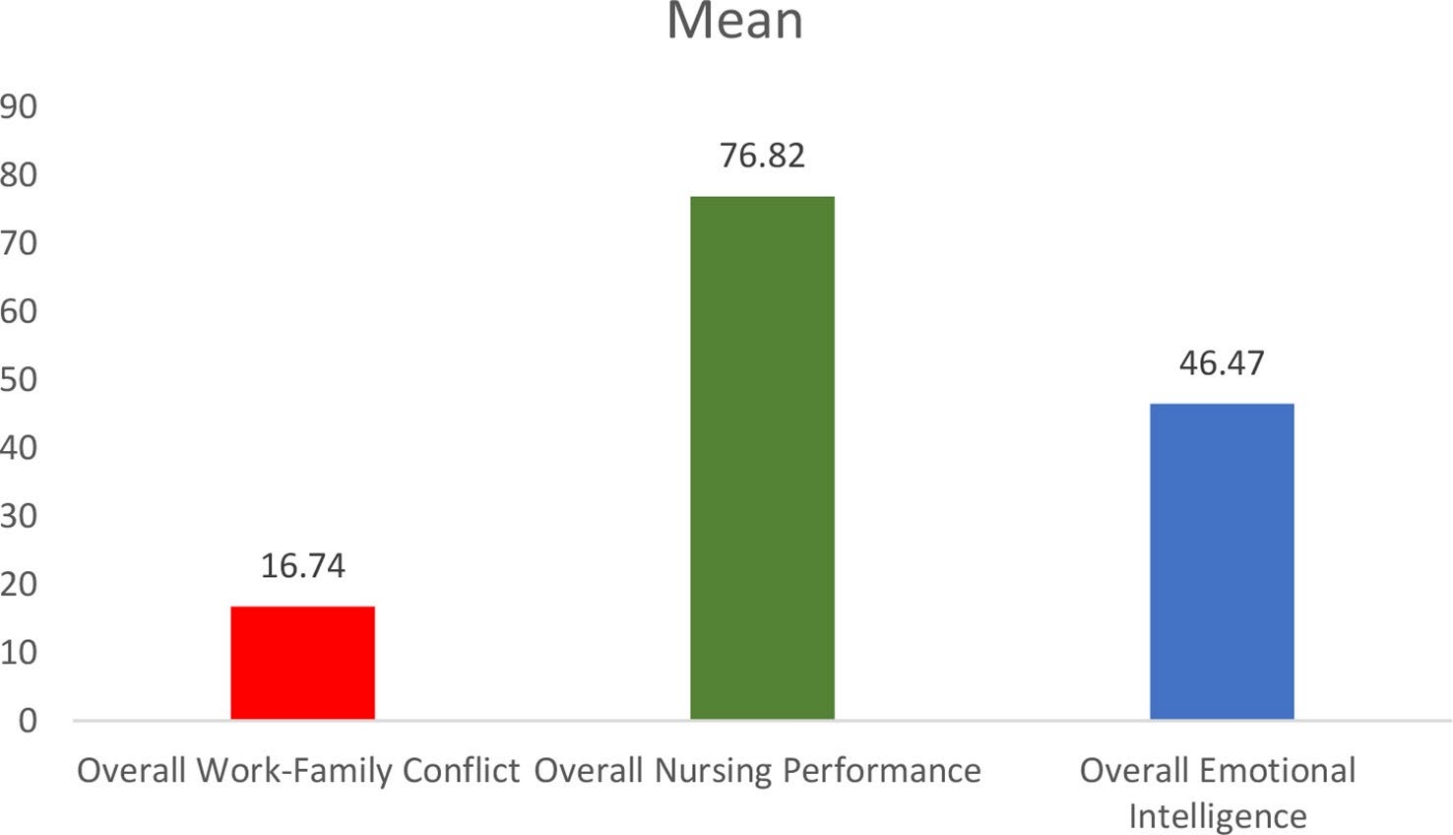
Mean values of the studied variables among nurses

Table 2; Fig. 2 present the descriptive analysis of the studied variables. Regarding WFC, the overall mean score is 16.74 with a standard deviation of 5.67, placing it in the low to moderate range (scale range: 10–50). This indicates that nurses experience moderate levels of conflict between their work and family responsibilities. Breaking it down further, the Family-Work Conflict subscale shows a slightly higher mean score of 11.76 (SD = 4.38), compared to the Work-Family Conflict subscale, which has a mean of 10.86 (SD = 4.10). This suggests that, on average, nurses face slightly more conflict from family responsibilities impacting their work than from work responsibilities affecting their family life.

For nursing JP, the overall mean score is 76.82 with a standard deviation of 26.49, which falls into the low to moderate range (scale range: 52–208). This indicates that, on average, nurses perform at a moderate level. Among the JP dimensions, Teaching/Collaboration scores the highest with a mean of 24.99 (SD = 10.43), followed by Interpersonal Relations with a mean of 23.69 (SD = 12.21). These scores suggest strengths in teamwork and communication among the nurses. However, lower mean scores are seen in Leadership, with a mean of 10.20 (SD = 4.72), and Planning/Evaluation, which has a mean of 12.82 (SD = 7.19). This indicates potential areas where performance could be improved. The Critical Care dimension scores a mean of 14.42 (SD = 5.45), and Professional Development has a mean of 15.30 (SD = 5.33), suggesting moderate engagement in these areas. The variability in scores across dimensions highlights the diverse range of JP levels among the nursing staff, with particular strengths in collaboration and interpersonal relations, but relatively lower performance in leadership roles.

The overall mean score for EI is 46.47 with a standard deviation of 19.28, which falls within the low to moderate range (scale range: 16–112). This suggests that, on average, nurses exhibit relatively moderate levels of EI, with significant variability in individual scores. Among the EI dimensions, the highest mean score is in Use of Emotion (UOE), which averages 13.42 (SD = 7.35), followed by Self-Emotions Appraisal (SEA) with a mean of 12.09 (SD = 7.50). The dimensions of the Others-Emotions Appraisal (OEA) and Regulation of Emotion (ROE) scores are slightly lower, with means of 11.87 (SD = 6.14) and 11.76 (SD = 6.88), respectively. These findings suggest that, while nurses exhibit some emotional intelligence capabilities, there is considerable room for development, particularly in the areas of self and others’ emotional appraisal, as well as emotion regulation.

**Table 2:** Descriptive analysis of nurses’ perceived WFC, JP, and EI

Dimensions	Mean	SD
**Overall Work-Family Conflict** ^a^	16.74	5.67
Work-Family Conflict Scale	10.86	4.10
Family-Work Conflict Scale	11.76	4.38
**Overall Nursing Performance** ^b^	76.82	26.49
Leadership	10.20	4.72
Critical Care	14.42	5.45
Teaching/Collaboration	24.99	10.43
Planning/Evaluation	12.82	7.19
Interpersonal Relations	23.69	12.21
Professional Development	15.30	5.33
**Overall Emotional Intelligence** ^c^	46.47	19.28
Self-Emotions Appraisal (SEA)	12.09	7.50
Others-Emotions Appraisal (OEA)	11.87	6.14
Use of Emotion (UOE)	13.42	7.35
Regulation of Emotion (ROE)	11.76	6.88

**Notes**:

a. 5-point Likert scale, Low (10–22), Moderate (23–35), High (36–50)

b. 4-point Likert scale, Low (52–104), Moderate (105–156), High (157–208)

c. 7-point Likert scale, Low (16–39), Moderate (40–75), High (76–112)

### Regression analysis among work-family conflict (WFC), job performance (JP), and emotional intelligence (EI)

Table 3; Fig. 1 illustrate the regression analysis between WFC as a predictor and both JP and EI. The analysis reveals that WFC has a statistically significant negative impact on JP (β = -0.08, *p* < 0.01). This suggests that higher levels of WFC are associated with lower JP. However, the very low R² value of 0.01 indicates that WFC explains only 1% of the variance in JP, highlighting a minimal direct impact.

In contrast, the relationship between WFC and EI was found to be non-significant (β = -0.02, *p* = 0.35). This indicates that WFC does not significantly affect EI, as supported by the R² value of 0.00, showing that WFC does not account for any variance in EI. This result partially supports Hypothesis 1.

Importantly, EI had a significant positive mediating effect on JP (β = 0.47, *p* < 0.01). This result suggests that higher levels of EI are associated with improved JP. The R² value of 0.22 shows that EI explains 22% of the variance in JP, indicating a moderate but meaningful effect. This finding underscores the importance of EI as a predictor of JP.

**Fig. 3:**
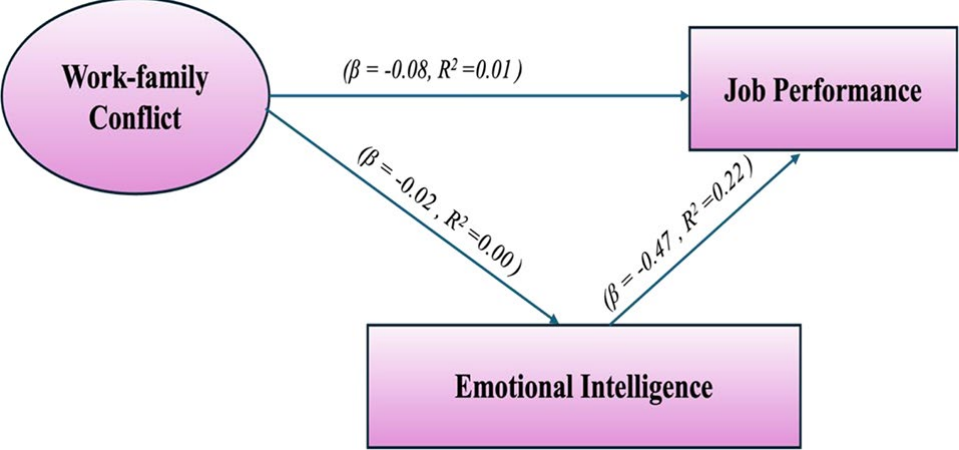
Standardized coefficients for the effect of WFC on nurses’ JP mediated by EI

As shown in Fig. 3, EI partially mediates the relationship between WFC and JP, further supporting Hypothesis 2. The model fit statistics indicate a generally good fit for the path model, with a Chi-Square (χ²) value of 15.40, a CFI of 0.98, and a Root Mean Square Error of Approximation (RMSEA) of 0.07.

**Table 3:** Path analysis among Work-Family conflict (WFC), job performance (JP), Emotional intelligence (EI)

Variables Path	β	*R* ^2^	S.E.	t-value	*P*
Work-Family Conflict → Job Performance	-0.08	0.01	0.03	-2.67	< 0.01*
Work-Family Conflict → Emotional Intelligence	-0.02	0.00	0.05	-0.40	0.35
Emotional Intelligence → Job Performance	0.47	0.22	0.05	9.40	< 0.01*

Note. R^2^ = regression coefficient; Model fit parameters: Chi-Square (χ²):15.40, Comparative Fit Index (CFI):0.98, Root Mean Square Error of Approximation (RMSEA):0.07, * p significant ≤ 0.05

### Demographic characteristics and studied variables

The analysis reveals that nurses’ demographic characteristics did not show significant effects on WFC, JP, or EI. See supplementary Table 1 for more details.

## Discussion

This research investigated the interconnections between WFC, JP, and EI among nursing professionals in Saudi Arabia. Furthermore, it analyzed the mediating influence of EI on the relationship between WFC and JP, contributing valuable insights into how these factors interact in the demanding nursing profession. The results indicated that nurses generally reported moderate levels of EI, WFC, and JP. These findings suggest that while nurses possess some emotional regulation abilities and maintain moderate job performance, they still face notable challenges in balancing work and family demands. The moderate levels of these variables are consistent with the challenging and emotionally demanding nature of nursing, especially in hospital settings with staffing shortages. Nurses often face emotional and physical exhaustion due to long hours and the complex care they provide [41]. The observed moderate levels of WFC among the nurses are consistent with studies conducted globally [5, 42], due to the emotional and physical demands of their roles. This moderate level of conflict reflects the high-stress environments in which healthcare professionals operate, especially in systems with chronic staffing shortages like Saudi Arabia. In Chain, Wu et al. [43] also highlighted similar patterns, noting that WFC is a prevalent issue due to the long working hours and the dual pressure of managing professional and family responsibilities.

In examining Hypothesis 1, which proposed a negative relationship between WFC and JP, the results confirmed that higher levels of WFC are associated with lower job performance. This finding is consistent with previous research [20, 21]. The negative impact of WFC on job performance is well-documented in the literature. When the obligations associated with one’s professional responsibilities and familial duties are incongruent, individuals may encounter psychological distress, leading to diminished job focus and performance [44]. This is particularly true in emotionally taxing professions such as nursing, where the overlap of personal stress with professional duties can lead to burnout and decreased job efficiency [5]. This suggests that WFC creates emotional and psychological strain, making it difficult for nurses to perform their duties effectively.

Moreover, the findings also confirmed Hypothesis 2, which suggested that EI mediates the relationship between WFC and JP. This result might be attributed to the ability to assess and control one’s emotions and understand others’ emotions, equipping nurses to handle workplace stress more effectively. Nurses with higher levels of EI demonstrated better job performance, even when experiencing moderate levels of WFC [25, 26]. This result highlights the importance of emotional regulation and resilience in mitigating the effects of WFC and work stress. This finding is supported by a previous study [9] that found nurses with higher EI were better equipped to manage work-related stress and maintain job performance despite facing WFC. Similarly, Hussain et al. [39] reported that nurses with high EI experienced fewer stressors, which allowed them to maintain higher job performance. This emphasized the role of EI in improving job performance by moderating the negative effects of stress from different resources, such as WFC. Hence, developing EI could be a critical intervention to help nurses cope with WFC and improve their job performance, especially in high-demand healthcare environments.

## Limitations

This study provides a meaningful interpretation of the relationship between work-family conflict (WFC) and job performance (JP), highlighting the mediating role of emotional intelligence (EI) among staff nurses in a Saudi hospital. However, several limitations should be acknowledged. First, the cross-sectional design limits the ability to infer causality between variables. A longitudinal design would better capture temporal changes and provide stronger evidence of directionality between WFC, EI, and JP.

Second, the reliance on self-reported questionnaires introduces potential response bias and social desirability effects. Although validated tools were used, the subjective nature of self-assessment may have influenced responses. Future studies should consider using multi-source assessments (e.g., peer or supervisor ratings of performance) or a mixed-methods approach to triangulate findings. Third, the study was conducted in a single governmental hospital in Riyadh. This limits generalizability to other healthcare settings, particularly private or rural hospitals, or institutions in other regions of Saudi Arabia and beyond. Future research should expand to a multicenter or national sample to increase the external validity of the results.

Fourth, while the study found that EI mediates the relationship between WFC and JP, the explained variance in JP was modest. This suggests that other influential factors—such as leadership style, work environment, organizational culture, and support systems—may also play a critical role and warrant further investigation. Finally, although the findings support the potential value of EI development, no intervention was tested. Future interventional studies should explore the impact of EI training programs on nurse performance and stress management to assess the practical benefits of enhancing emotional competencies in clinical settings.

## Conclusion

This study investigated the effect of work-family conflict (WFC) on staff nurses’ job performance (JP) and examined the mediating role of emotional intelligence (EI). The findings confirmed that WFC negatively influences JP, indicating that increased conflict between professional and personal roles can hinder nurses’ effectiveness. Importantly, EI emerged as a significant mediator in this relationship. Nurses with higher EI levels demonstrated better job performance despite experiencing WFC. This suggests that EI serves as a protective factor, enabling nurses to manage emotional demands and maintain performance under stress. These results underscore the need to foster emotional intelligence among nursing staff as a strategic approach to mitigate the adverse effects of WFC and enhance the quality of care delivered.

## Implications

This study highlights emotional intelligence (EI) as a critical mediator in the relationship between work-family conflict (WFC) and job performance (JP) among staff nurses. The findings carry important implications for nursing education, healthcare practice, and policy, particularly within the Saudi Arabian context. These implications underscore the importance of emotionally intelligent systems, not just individuals. Aligning organizational structures, educational curricula, and national policies around emotional competence and work-life integration is essential for sustaining a high-performing nursing workforce in Saudi Arabia.

Given the diverse and multicultural nature of the nursing workforce in Saudi Arabia, with a high proportion of expatriate nurses and varying cultural norms around family roles, tailored interventions are essential. Nurses working long shifts in high-pressure settings, especially in large tertiary hospitals, often experience competing demands between work and family. Incorporating EI training into both undergraduate and continuing professional education programs could help nurses develop self-regulation, empathy, and emotional control—skills necessary for maintaining performance despite stress. For example, including EI-focused simulations and reflective exercises in clinical education would allow students to practice emotional coping in realistic scenarios.

*Healthcare policymakers* in Saudi Arabia should prioritize initiatives that support work-life balance. These could include offering flexible scheduling options, childcare services for shift-working mothers, and employee assistance programs (EAPs) that include mental health support. Implementing such policies could help reduce the emotional strain associated with WFC, particularly in governmental hospitals where staffing shortages and heavy workloads are common.

*For nurse managers*, EI should be considered a core leadership competency. Integrating EI assessments into recruitment and promotion processes, especially for supervisory roles, could ensure that leaders are equipped to manage diverse teams effectively and support staff facing WFC. Practical strategies include regular training workshops on managing interpersonal conflict, communication under stress, and emotionally intelligent leadership.

*Healthcare organizations* in Saudi Arabia must also address systemic contributors to WFC, such as chronic understaffing and limited autonomy in scheduling. Regular monitoring of staff workload and family-related stressors, through feedback systems or wellbeing surveys, could inform more responsive human resources policies. For example, allowing partial remote work or part-time options for nurses with family responsibilities could reduce turnover and improve performance outcomes.

*On the educational front*, nursing colleges should embed EI development into curricula. This can be done through stand-alone modules, mentorship models, and clinical debriefings that focus on emotional regulation and interpersonal sensitivity. Given the evolving demands on Saudi nurses, early exposure to these skills can foster resilience and enhance readiness for clinical practice.

Finally, *future research* should explore WFC and EI across different healthcare sectors and regions in Saudi Arabia. Comparative studies between public and private hospitals, or across rural and urban facilities, may reveal contextual factors influencing the EI-WFC-JP dynamic. Intervention studies that test the effectiveness of structured EI development programs—such as mindfulness training, peer coaching, or resilience workshops—would offer evidence-based solutions to strengthen nurse performance and well-being.

## Electronic supplementary material

Below is the link to the electronic supplementary material.

Supplementary Material 1

## Data Availability

The data are available from the authors upon reasonable request.

## Declarations

### Declaration of Helsinki-Research

Throughout the study, the ethical principles of the Declaration of Helsinki were followed.

## Abbreviations

BSN: Bachelor of Science in Nursing
CFI: Comparative Fit Index
EAP: Employee Assistance Program
EI: Emotional Intelligence
FWC: Family–to–Work Conflict
IRB: Institutional Review Board
JP: Job Performance
KAIMRC: King Abdullah International Medical Research Center
OEA: Others–Emotions Appraisal
RMSEA: Root Mean Square Error of Approximation
ROE: Regulation of Emotion
SEA: Self–Emotions Appraisal
SPSS: Statistical Package for the Social Sciences
UOE: Use of Emotion
WFC: Work–Family Conflict
WLEIS: Wong and Law Emotional Intelligence Scale
