# Supplementary material for: The effect of work-family conflict on staff nurses’ job performance: the mediating role of emotional intelligence
Source: BMC Nurs. 2025 May 30;24:614. doi: 10.1186/s12912-025-03280-w (PMC12124067; doi:10.1186/s12912-025-03280-w)
Supplement: Supplementary file 1 — Supplementary Material 1 [file 12912_2025_3280_MOESM1_ESM.docx]

### **Supplementary Table 1.** *Perceived Differences in Job Performance, Emotional Intelligence, and Work-Family Conflict According to Demographic Data*

| **Demographic/ Academic Data** | **Overall Job Performance (Mean ± SD)** | **Overall Emotional Intelligence (Mean ± SD)** | **Overall Work-Family Conflict (Mean ± SD)** |
| --- | --- | --- | --- |
| **Age** |  |  |  |
| < 21 years | 72.34 ± 25.10 | 44.20 ± 18.50 | 15.90 ± 5.20 |
| 21-30 years | 74.22 ± 26.50 | 45.30 ± 19.40 | 16.20 ± 5.30 |
| 31-40 years | 78.11 ± 27.30 | 46.80 ± 20.10 | 16.70 ± 5.50 |
| 41-50 years | 76.50 ± 26.40 | 47.20 ± 20.00 | 17.10 ± 5.80 |
| > 50 years | 77.40 ± 26.70 | 46.90 ± 19.70 | 17.20 ± 5.60 |
| **F (p-value)** | 0.78 (0.61) | 0.82 (0.53) | 0.87 (0.51) |
| **Marital Status** |  |  |  |
| Single | 75.80 ± 26.20 | 46.50 ± 19.50 | 16.40 ± 5.50 |
| Married, no kids | 76.00 ± 26.70 | 46.20 ± 19.80 | 16.50 ± 5.60 |
| Married with kids | 77.30 ± 27.10 | 47.00 ± 20.00 | 17.10 ± 5.70 |
| Widow | 72.40 ± 25.50 | 44.90 ± 18.90 | 15.80 ± 5.30 |
| **F (p-value)** | 1.24 (0.16) | 1.15 (0.21) | 1.03 (0.42) |
| **Educational Level** |  |  |  |
| Diploma | 74.50 ± 25.70 | 45.70 ± 19.20 | 16.10 ± 5.40 |
| BSN | 77.60 ± 26.80 | 47.20 ± 19.90 | 16.90 ± 5.50 |
| Master’s Degree | 78.20 ± 27.10 | 47.90 ± 20.30 | 17.20 ± 5.60 |
| **F (p-value)** | 0.77 (0.89) | 0.81 (0.86) | 0.81 (0.24) |
| **Years of Experience** |  |  |  |
| < 1 year | 71.80 ± 24.90 | 44.80 ± 18.70 | 15.80 ± 5.20 |
| 1-5 years | 73.90 ± 25.60 | 45.90 ± 19.60 | 16.40 ± 5.50 |
| 6-10 years | 76.20 ± 26.80 | 46.70 ± 20.10 | 16.80 ± 5.60 |
| > 10 years | 78.40 ± 27.20 | 47.50 ± 20.00 | 17.10 ± 5.70 |
| **F (p-value)** | 0.65 (0.97) | 0.79 (0.90) | 0.57 (0.99) |
